# Regulation of N6-Methyladenosine in the Differentiation of Cancer Stem Cells and Their Fate

**DOI:** 10.3389/fcell.2020.561703

**Published:** 2020-09-22

**Authors:** Ya Xu, Jing Liu, Wen-Jia Chen, Qian-Qian Ye, Wen-Tian Chen, Chun-Lan Li, Hua-Tao Wu

**Affiliations:** ^1^Department of General Surgery, The First Affiliated Hospital of Shantou University Medical College, Shantou, China; ^2^Changjiang Scholar’s Laboratory/Guangdong Provincial Key Laboratory for Diagnosis and Treatment of Breast Cancer, Shantou University Medical College, Shantou, China; ^3^Department of Physiology/Cancer Research Center, Shantou University Medical College, Shantou, China

**Keywords:** m6A, stem cell, differentiation, cancer, hematopoietic

## Abstract

N6-methyladenosine (m6A) is one of the most common internal RNA modifications in eukaryotes. It is a dynamic and reversible process that requires an orchestrated participation of methyltransferase, demethylase, and methylated binding protein. m6A modification can affect RNA degradation, translation, and microRNA processing. m6A plays an important role in the regulation of various processes in living organisms. In addition to being involved in normal physiological processes such as sperm development, immunity, fat differentiation, cell development, and differentiation, it is also involved in tumor progression and stem cell differentiation. Curiously enough, cancer stem cells, a rare group of cells present in malignant tumors, retain the characteristics of stem cells and play an important role in the survival, proliferation, metastasis, and recurrence of cancers. Recently, studies demonstrated that m6A participates in the self-renewal and pluripotent regulation of these stem cells. However, considering that multiple targets of m6A are involved in different physiological processes, the exact role of m6A in cancer progression remains controversial. This article focuses on the mechanism of m6A and its effects on the differentiation of cancer stem cells, to provide a basis for elucidating the tumorigenesis mechanisms and exploring new potential therapeutic approaches.

## Introduction

N6-methyladenosine (m6A) is one of the most common internal modifications in eukaryotic mRNAs and non-coding RNAs including long non-coding RNAs (lncRNA), microRNAs (miRNAs), ribosomal RNAs (rRNAs), small nuclear RNAs (snRNAs), and transfer RNAs (tRNAs) (Bokar et al., 1997; Fu et al., 2014). This dynamic and reversible modification was first discovered in the 1970s, and it involves three types of molecules: methyltransferases, demethylases, and methylated binding proteins (Bokar et al., 1997; Fu et al., 2014). Recent emerging studies suggested that m6A is not only involved in the normal physiological processes but also associated with the occurrence of and development of multiple cancers (Deng et al., 2018; Lai et al., 2018; Zhang et al., 2018; Wei et al., 2019).

Stem cells including totipotent stem cells (TSCs), pluripotent stem cells (PSCs), and unipotent stem cells (USCs) have a strong ability of self-renewal, proliferation, and differentiation (Bozdag et al., 2018; Sotthibundhu et al., 2018). TSCs can be differentiated into full organisms, the same way as embryonic stem cells (ESCs) do (Bozdag et al., 2018). PSCs, which are also called mesenchymal stem cells (MSCs), have the potential to differentiate either into a variety of cellular tissues or into different cells of a certain tissue type, like hematopoietic stem cells (HSCs) (Kashima et al., 2018). USCs can only differentiate into one or two closely related cell types, such as the mammary stem cells (Lilja et al., 2018). The stemness of stem cells is determined by the presence of certain protein molecules, and the expression of these molecules is mainly controlled through DNA methylation, histone acetylation, and miRNAs (Moussaieff et al., 2015; Shim and Nam, 2016; Ran et al., 2017; Wang et al., 2017). Based on the rapid development of research strategies and technologies, many stem cells core pluripotency factors have been identified, including Octamer-binding transcription factor 4 (OCT4), SRY-box 2 (SOX2), and NANOG (Hu et al., 2008; Leis et al., 2012; Iv Santaliz-Ruiz et al., 2014). It has been demonstrated that m6A methylation is indispensable for the pluripotency and differentiation of ESCs and HSCs (Batista et al., 2014; Wang et al., 2014; Vu et al., 2017; Li et al., 2018; Weng et al., 2018). These biological properties of stem cells make them a research hotspot, whether in basic scientific research or in clinical medicine research.

In malignant tumors, it has been suggested that some cancer cells such as cancer stem cells (CSCs) have similar biological characteristics as those of stem cells, such as self-renewal ability and multiple differentiation potential, thereby producing heterogeneous tumor cells (Reya et al., 2001; Prasetyanti and Medema, 2017). In 1994, through specific cell surface markers, Lapidot et al. isolated a type of cell with self-renewal and maintenance of malignancy properties from leukemia cells, named as acute myelogenous leukemia stem cells (LSCs); this was the first confirmation of the existence of CSCs (Lapidot et al., 1994). Currently, with infinite proliferation abilities, the important role of CSCs in the occurrence and development of malignant tumors, such as tumor survival, proliferation, metastasis, and recurrence, was confirmed (Reya et al., 2001; Chang, 2016; Pan et al., 2018). Identification and elimination of CSCs in malignant tumors have become a new strategy for treatment. The differentiation of CSCs is controlled by many factors such as abnormal activation of the PI3K/Akt/mTOR axis, Wnt and Notch signaling pathways, and adhesion molecules such as cadherin and integrin that mediate the anchoring of stem cells to their niche (Lin, 2002; Indrayani, 2018; Venkatesh et al., 2018). Recent studies demonstrated that m6A participates in the self-renewal and pluripotent regulation of CSCs (Zhang C. et al., 2016, 2017; Zhang S. et al., 2017). However, as the multiple targets of m6A are involved in different physiological processes, the role of m6A remains controversial. Therefore, this review focuses on the mechanism of m6A and its role in the differentiation of stem cells and CSCs to determine their roles in malignant tumors.

## m6A Processes and Their Functions

**FIGURE 1:**
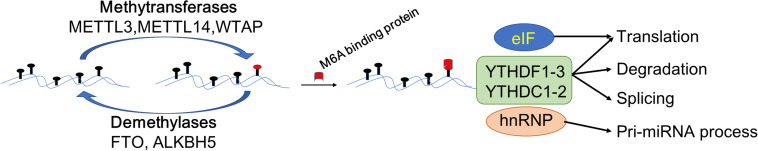
Schematic elucidation of m6A modification in the regulation of gene expression. The m6A modification is catalyzed by methyltransferases METTL3/14, Wilms tumor 1-associating protein (WTAP). The demethylases fat mass and obesity-associated protein (FTO) and AlkB homolog 5 (FTO/ALKBH5) demethylated the bases modified by m6A. Methylated reader proteins recognize the mRNA modified by m6A, thereby activating downstream pathways by different reader proteins. After modified by methyltransferases, eIF3 proteins promote mRNA translation. HNRNPA2B1 regulates the processing of the pre-miRNA and pri-miRNA. Further, YTHDF1-3, YTHDC1-2 can regulate the processes of RNA translation, degradation, and splicing.

The m6A modification is catalyzed by an unidentified methyltransferase complex containing at least one subunit, METTL3. In some cases, it can be read and erased by reader proteins and demethylases (Roundtree et al., 2017; Figure 1). Increasing evidence suggests that m6A modification is misregulated in human cancers and may be ideal targets of cancer therapy (Barbieri and Kouzarides, 2020). The m6A modification affects the pathogenesis of multiple diseases and cancers, not only by affecting coding RNAs but also by affecting non-coding RNAs, such as microRNAs, lncRNAs and circRNAs (Fazi and Fatica, 2019; Zhang et al., 2020).

### Methyltransferases

Methyltransferases function as enzymes that act downstream of mRNA adenylate undergoing m6A modification, including methyltransferase-like 3 (METTL3), methyltransferase-like 14 (METTL14), Wilms tumor 1-associating protein (WTAP), and KIAA1492 (Schwartz et al., 2014). Previously, it was thought that METTL3 and METTL14 are methyltransferases involving the formation of a heterodimer complex that functions in cellular m6A deposition on mammalian nuclear RNAs (Liu et al., 2014). Recently, it was found that METTL3 plays the role of the main enzyme of methyltransferases, whereas METTL14 promotes the binding of METTL3 to the targeted RNA (Wang et al., 2016). WTAP is responsible for recruiting the METTL3-METTL14 heterodimer complex into nuclear speckles (Wu et al., 2018). WTAP and METTL3-METTL14 are co-localized in the nuclear speckles where they participate in the process of RNA splicing (Liu et al., 2014). Further, it was reported that KIAA1492 is another core protein belonging to methyltransferases, which is also localized in the nuclear speckles; however, its function is unclear (Schwartz et al., 2014). Importantly, the above-mentioned methyltransferases do not work in isolation but rather form a complex in which they work together to catalyze the respective modifications on downstream target RNAs (Wang et al., 2016).

### Demethylases

Demethylases perform a reverse process to that described above and demethylate the mRNA modified with m6A and hence also known as an eraser. Thus far, demethylases included two reported proteins: fat mass and obesity-associated protein (FTO) and AlkB homolog 5 (ALKBH5)(Jia et al., 2011; Zheng et al., 2013). FTO, belonging to the AlkB family of non-heme Fe (II)/dioxygenases, was the first identified demethylase of m6A in RNAs (Jia et al., 2011). FTO contributes to the regulation of mRNA alternative splicing by modulating m6A levels (Batista et al., 2014; Zhao et al., 2014). ALKBH5, also belonging to the AlkB family, has been identified as a demethylase for m6A modification of RNAs (Zheng et al., 2013). ALKBH5 regulates mRNA export, RNA metabolism, and assembly of mRNA processing factors in nuclear speckles (Zheng et al., 2013).

### Methylated Reader Proteins

The reversible chemical modification requires the recognition of the m6A-modified RNAs by reader proteins such as YTH domain proteins, nuclear heterogeneous ribonucleoprotein (hnRNP), and eukaryotic initiation factors (eIF), which are involved in the translation, degradation, and miRNA processing of downstream targets in the pathway (Wang X. et al., 2014). The fate of m6A-RNA is varied and even “contradictory.” For example, in different target RNAs, m6A modification can promote both the translation and degradation processes of mRNA, which is determined by the m6A reading protein “reader” (Figure 1). Previous studies showed that recognition by YTHDF1/3 promoted the translation process of mRNA (Shi et al., 2017; Han et al., 2020), whereas recognition by YTHDF2 induced mRNA degradation process (Zhao et al., 2017; Lai et al., 2018). Diversely, eIF3 proteins are mainly bound to the 5′ untranslational region (5′-UTR) of RNAs to promote mRNA translation (Skabkin et al., 2015), while hnRNPA2/B1, which is one of the hnRNP proteins, recognizes target m6A-RNAs, activates the downstream pathway of the pri-miRNA, and regulates the processing of the pre-miRNA (Alarcon et al., 2015).

### Functions of the m6A Process

#### m6A Participates in Physiological Activities

The m6A modification influences the downstream pathways by regulating the fate of an RNA transcript, processing, splicing, degradation, or translation, whether mRNA or non-coding RNA(Fazi and Fatica, 2019; Zhang et al., 2020). It plays an important role in various biological processes at different levels of the m6A modification of RNA, such as circadian clock (Fustin et al., 2013, 2018; Zhong et al., 2018), DNA damage response (Xiang et al., 2017; Zlotorynski, 2017), neural function regulation (Poeck et al., 2016; Zhang F. et al., 2017), drosophila sex determination (Haussmann et al., 2016; Poeck et al., 2016), and embryonic development (Kwon et al., 2019).

For example, PER2 and Bmal1 were discovered as the clock genes that control the pace of our daily lives to maintain the human circadian clock (Lowrey and Takahashi, 2011). Casein Kinase 1 Delta mRNA (Ck1δ) encodes a critical kinase that controls circadian rhythms by enhanced translation of PER2, which is negatively regulated by m6A (Zhong et al., 2018). When m6A is inhibited, CK1δs levels are increased, and the increased stabilization of the PER2 protein, as a result, leads to a slower clock (Zhong et al., 2018). Zhong et al. (2018) also found that the m6A modification was involved in the regulation of the circadian clock through the clock gene Bmal1 (Fustin et al., 2018). Bmal1 affects the levels of m6A modification and controls the expression of PPARα to regulate lipid metabolism. These findings revealed a new way by which the circadian clock regulates metabolism. Another study found that m6A modulates sex determination in drosophila (Haussmann et al., 2016). As Sxl (Sex-lethal) is a switch gene involved in sex determination, the m6A modification of the pre-mRNA of Sxl, affected its selective splicing and thereby the regulation of drosophila sex development (Haussmann et al., 2016).

#### m6A Participates in the Pathological Processes of Diseases

The m6A modification also causes diseases such as neurodevelopmental delay (Li H.B. et al., 2017; Yoon et al., 2017), immunodeficiency (Li H.B. et al., 2017), and male infertility (Zheng et al., 2013; Yang et al., 2016). Based on current evidence, the findings and investigations on m6A function provide a new direction for the treatment of these diseases. The m6A modulates murine spermatogenesis; after the inactivation of m6A methyltransferases, the level of m6A modification significantly reduced, which could lead to sperm formation disorder (Li H.B. et al., 2017). m6A methylation is also involved in regulating testosterone synthesis in Leydig cells (LCs); the study on m6A methylation provides a new direction for the treatment of azoospermia and oligospermia (Chen et al., 2020). Li H.B. et al. (2017) found that m6A modification controlled T cell homeostasis by targeting the IL-7/STAT5/SOCS pathways. After the knockout of METTL3, m6A modification in T cells decreased, thus impairing their ability to differentiate. Consequently, these T cells could not cause autoimmune diseases, providing a new way to alleviate autoimmune diseases with drugs that target m6A modification (Li H.B. et al., 2017).

#### m6A Participates in the Development of Malignant Tumors

It was not surprising, therefore, to find that m6A modification was involved in the occurrence and development of different types of malignant tumors (Deng et al., 2018; Lai et al., 2018; Wei et al., 2019). m6A modification affects tumor proliferation, differentiation, tumorigenesis, invasion, and metastasis by regulating proto-oncogenes and tumor suppressor genes. The translation of the m6A modified gene was changed, which affected the development and progression of the tumor. For example, in lung cancer, METTL3 promotes cell growth and leads to cancer by increasing the expression of EGFR and TAZ (Lin et al., 2016). In human hepatocellular carcinoma, knockdown of METTL3 decreased SOCS2 mRNA modification and increased SOCS2 mRNA expression, suppressing the progression of liver cancer (Chen et al., 2018). Recently, it has been demonstrated that m6A methylation participates in the self-renewal and pluripotent regulation of stem cells, even in CSCs (Zhang S. et al., 2017; Wu et al., 2018). To explore the underlying role of m6A in the differentiation of CSCs, the next part of this review focuses on research related to m6A function in CSCs.

## Role of m6A in the Differentiation of CSCs

### CSCs in Leukemia

#### The Role of Leukemia Stem Cells (LSCs) in the Occurrence of Myeloid Leukemia

Typically, HSCs differentiate into myeloid progenitors and eventually mature myeloid cells (Nishikii et al., 2017). Dysregulation of this process results in the development of diseases such as acute myeloid leukemia (AML), an aggressive clonal disease of abnormal HSCs, and primitive progenitors that blocks their myeloid differentiation to generate self-renewing leukemia stem cells (LSCs) (Testa, 2011). Furthermore, the presence of leukemia stem/initiating cells (LSCs/LICs) can lead to the occurrence or relapse of myeloid leukemia, which is likely to be a major cause of drug-resistant disease and relapse in AML patients (ten Cate et al., 2010).

#### m6A in the Processes of Hematopoiesis

Recently, it has been revealed that m6A participates in the process of endothelial hematopoietic transition (EHT), which is the mechanism underlying HSCs generation (Thambyrajah et al., 2016). In invertebrates, the Notch signaling pathway is critical to the development of hematopoietic stem and progenitor cells (HSPCs) during embryogenesis (Robert-Moreno et al., 2008). Zhang et al. demonstrated that G protein-coupled receptor 183 (Gpr183) signaling repressed Notch signaling before the onset of EHT, serving as an indispensable switch for HSPC emergence, and the inhibition of Gpr183 abolished HSPC emergence by significantly upregulating Notch signaling (Zhang et al., 2015). Another investigation revealed that in zebrafish, the stability of notch 1 was mediated by METTL3 through m6A modification and recognized by YTHDF2 to maintain the balance of gene expression during the EHT process, thus regulating the fate of HSCs (Zhang C. et al., 2017). Generally, METTL14 can be suppressed by SPI1, which plays an essential role in generating early myeloid progenitors (Weng et al., 2018). As expected, the critical role of METTL3 and METTL14 in normal and malignant hematopoiesis was proved, and there is evidence that the expression levels of METTL3 and METTL14 are highly increased in HSPCs and decreased during normal differentiation (Vu et al., 2017; Weng et al., 2018).

To explore the physiological functions of YTHDF2, Li et al. used the conditional mouse model of *Ythdf2* knockout and found that the number of functional HSCs increased without skewing lineage differentiation or causing hematopoietic malignancies. This demonstrates the physiological functions of YTHDF2 in adult stem cell maintenance by regulating the stabilities of mRNAs critical for self-renewal of HSCs (Li et al., 2018).

#### m6A in the Processes of Leukemogenesis

**FIGURE 2:**
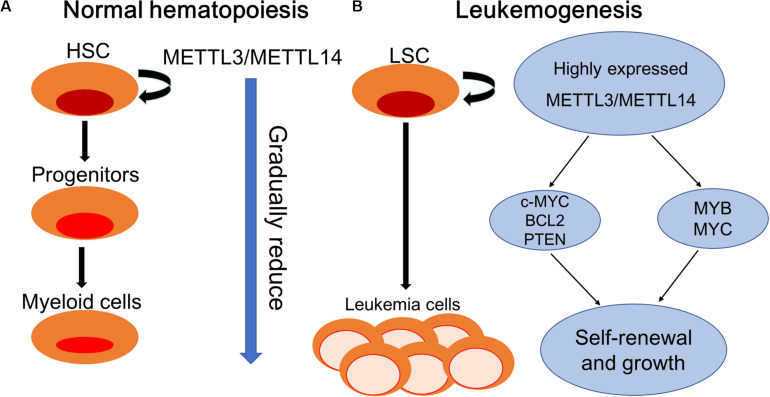
Schematic elucidation of normal hematopoiesis and leukemogenesis. **(A)** Normal hematopoiesis: hematopoietic stem cells (HSCs) differentiate into myeloid progenitors and eventually mature myeloid cells. METTL3/METTL14 are highly expressed in hematopoietic stem and progenitor cells (HSPCs) and are downregulated during normal differentiation. **(B)** Leukemogenesis: myeloid differentiation of HSPCs is blocked to produce self-renewing LSCs. METTL3/METTL14 are aberrantly expressed in leukemia stem cells (LSCs). METTL3 promotes the self-renewal and growth of LSCs by enhancing the translation of c-MYC, BCL2, and PTEN mRNAs. METTL14 promotes self-renewal and growth through the induction of MYB and MYC mRNA translation.

The proto-oncogenes MYB and MYC are reported to be overexpressed in many human malignant tumors including AML and contribute to disease progression by inhibiting differentiation and promoting self-renewal of AML cells (Gonda and Metcalf, 1984; Bahr et al., 2018). Ramsay et al. reported the aberrant expression of METTL14 in AML cells and its involvement in the regulation of the expression of MYB and MYC through m6A-based post-transcriptional regulation, indicating the critical role of METTL14 in the self-renewal of LSCs/LICs and development of AML (Ramsay and Gonda, 2008). Vu et al. (2017) demonstrated the oncogenic role of m6A in myeloid leukemia by promoting the translation of c-MYC, BCL2, and PTEN mRNAs (Figure 2).

For treating hematological disorders including cancer, transplantation of HSCs from human umbilical cord blood (hUCB) holds great application foreground but has restrictive uses because of limited numbers (Gincberg et al., 2018). In hematopoietic malignancies, the expression of YTHDF2 in leukemia patients was significantly increased, suggesting the potential promoting function of YTHDF2 in the occurrence and development of leukemia (Paris et al., 2019). In animal models, it was found that suppressing YTHDF2 expression can significantly inhibit the leukemia process and prolong the survival period of tumor-bearing mice, indicating that YTHDF2 is very important to the development of leukemia (Paris et al., 2019). Paris et al. (2019) pointed out that YTHDF2 was not essential for normal HSC function; however, increased expression of YTHDF2 was required for both initiation and propagation of AML, contributing to the integrity of LSC function by decreasing stabilities of m6A transcripts including the tumor necrosis factor receptor *Tnfrsf2*. Importantly, the upregulation of *Tnfrsf2* in *Ythdf2*-deficient LSCs primed malignant cells for apoptosis, predicting YTHDF2 as a potential therapeutic target in patients with AML to selectively inhibit LSCs and promote the expansion of HSCs (Paris et al., 2019).

### CSCs in Solid Tumors

In addition to the role of m6A in the regulation of the differentiation of normal hematopoietic process and leukemia hematopoietic process, recent evidence focuses on the role of m6A in regulating tumorigenesis in solid tumors by affecting the fate of CSCs (Table 1).

**TABLE 1:** The reported roles of m6A enzymes in CSCs of solid tumors.

**CSCs**	**m6A enzyme**	**Targets**	**Reported Function**	**References**
BCSC	ALKBH5	NANOG	Increase the percentage of BCSCs	Zhang C. et al., 2016; Zhang C. Z. et al., 2016
GSCs	METTL3	SOX2	Enhance radiation resistance	Visvanathan et al., 2018
	ALKBH5	FOXM1	Enhance self-renewal and tumorigenesis	Zhang S. et al., 2017
	FTO	ADAM19	Enhance GSC growth and self-renewal	Cui et al., 2017
CCSCs	YTHDF1	Wnt/β-catenin pathway	Enhance colonosphere self-renewal and suppresses differentiation	Bai et al., 2019

*CSCs, cancer stem cells; BCSCs, breast cancer cells; GSCs, glioblastoma stem cells; CCSCs, colorectal cancer stem cells.*

#### Breast Cancers

Breast CSCs (BCSCs), with their infinite proliferative ability through self-renewal and transient amplifying cells, play important roles in tumor growth, motility, invasion, metastasis, and resistance to chemotherapy (Beretov et al., 2018). Oskarsson et al. (2014) systematically reviewed the sources, niches, and vital pathways of metastatic stem cells and elucidated that metastasis in malignant tumors was powered and initiated by disseminated cancer cells with survival, self-renewal, dormancy, and reactivation abilities, namely, metastatic stem cells (MetSCs). Interestingly, the existence of BCSCs was originally described as of hematopoietic origin (Al-Hajj et al., 2003; Mani et al., 2008; Pece et al., 2010), and the MetSCs were capable of reinitiating distant tumor growth, independent of the origin or phenotypic characteristics of primary tumors (Oskarsson et al., 2014). Certain cytokines were proven to stimulate CSC features and that BCSC potential was promoted by transforming growth factor β (TGF-β) in synergy with the Wnt signaling pathway (Scheel et al., 2011). Zhang et al. (2009, 2013) found that abnormal CXCL12/IGF1 signaling and Src activities in patients with breast tumors predicted an increased risk of bone relapse. It is accepted that the phenotype of BCSCs is distinct and specified by the expression of core pluripotency factors including Kruppel-like factor 4 (KLF4), OCT4, SOX2, and NANOG (Hu et al., 2008; Yu et al., 2011; Leis et al., 2012; Iv Santaliz-Ruiz et al., 2014), providing potential effective therapeutic strategies for patients with breast cancer to eliminate BCSCs (Oskarsson et al., 2014).

**FIGURE 3:**
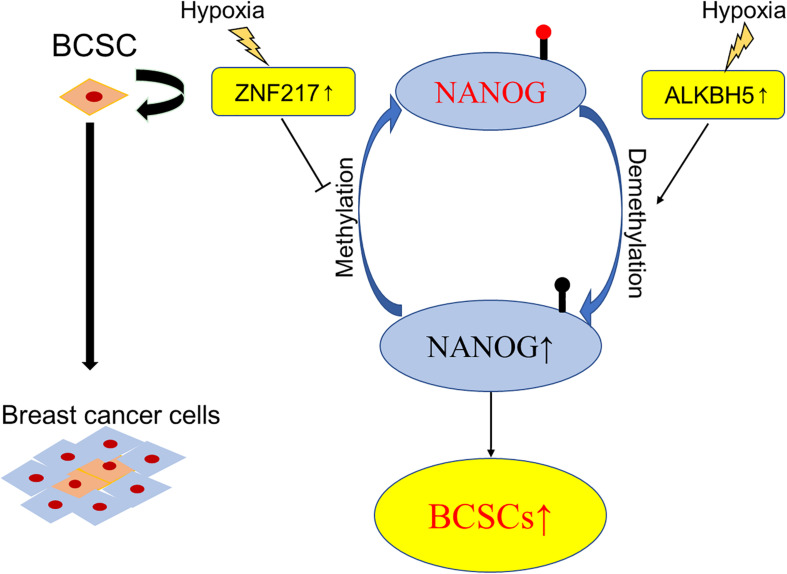
Schematic elucidation of m6A in BCSCs. Exposure of breast cancer cells to hypoxia can induce ALKBH5-mediated demethylation of NANOG mRNA, leading to increased expression of NANOG, and increasing the percentage of BCSCs.

It is accepted that breast cancer involves intratumoral regions under hypoxic conditions with activated hypoxia-inducible factors (HIFs) during the development process, and in response to hypoxia or chemotherapy, HIFs induce the BCSCs phenotype accordingly, which is implicated in resistance to chemotherapy, disease recurrence, and metastasis (Xiang and Semenza, 2019). Using animal models (Zhang C. et al., 2016) found that HIF-induced expression of ALKBH5, an m6A demethylase, promoted the BCSCs phenotype by demethylating and increasing the mRNA levels of NANOG, a pluripotency factor. Soon after, the same group demonstrated another molecular mechanism of HIF-induced pluripotency with BCSCs specification, namely, zinc finger protein 217 (ZNF217)-dependent inhibition of m6A methylation of NANOG and KLF4 (Zhang C. Z. et al., 2016). These findings verified the participation of m6A modification in the differentiation of BCSCs and provided novel therapeutic targets for breast cancer patients, especially in the hypoxic tumor microenvironment (Figure 3).

#### Glioblastoma (GBM)

Glioblastoma (GBM) is the most prevalent and lethal primary tumor in the brain, with invasion into the surrounding brain structures. Conventional therapeutic strategies include surgery, radiotherapy, and adjuvant chemotherapy (Stupp et al., 2009). Although targeted therapies or immunotherapies are reported to fight GBM to improve the survival and quality of life of GBM patients, an efficient cure was never achieved (Diaz et al., 2017). Lathia et al. (2015) systematically reviewed the role of CSCs in GBM and demonstrated that self-renewing and tumorigenic abilities of CSCs contributed to tumor initiation and therapeutic resistance.

Cui et al. (2017) verified the critical role of m6A modification in the self-renewal and tumorigenesis of glioblastoma stem cells (GSCs) by artificially modifying the expression of METTL3 or METTL14, the key components of the RNA methyltransferase complex, *in vitro* and *in vivo*. Knockdown of METTL3 or METTL14 enhances GSCs growth and self-renewal, in contrast, an FTO inhibitor suppresses the progression of GSC-initiated tumors (Cui et al., 2017). The abnormal expression of ALKBH5 was also detected in GSCs, to demethylate FOXM1 nascent transcript and enhance the expression of Forkhead box protein M1 (FOXM1) (Zhang S. et al., 2017). Interestingly, a long non-coding RNA FOXM1-AS (antisense to FOXM1) promotes the interaction between ALKBH5 and FOXM1 transcript and GSC tumorigenesis through the FOXM1 axis (Zhang S. et al., 2017). As the FOXM1 and adamalysin-19 (ADAM19) have proved to play oncogenic roles in malignant tumors (Nandi et al., 2018; Wang et al., 2019a), the above-mentioned evidence proved the oncogenic function of the m6A demethylases ALKBH5 and FTO in enhancing self-renewal and tumorigenesis through the regulation of FOXM1 and ADAM19, respectively (Cui et al., 2017; Zhang S. et al., 2017).

**FIGURE 4:**
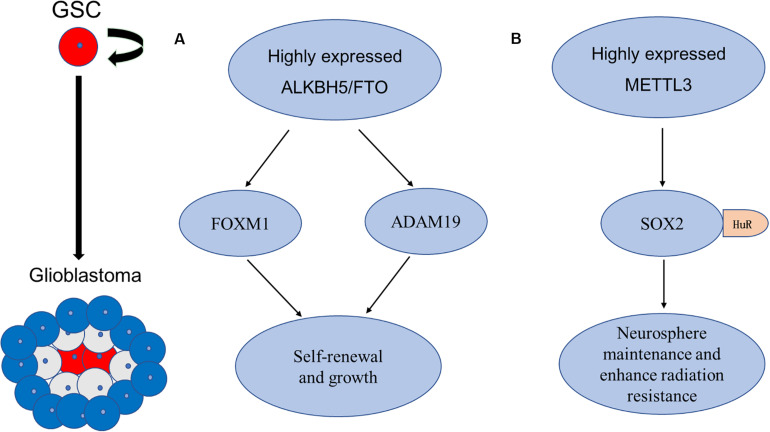
Schematic elucidation of m6A in GSCs. **(A)** Cui et al. and Zhang et al. found that RNA m6A demethylase ALKBH5 and FTO are aberrantly expressed in GSCs, and they enhance self-renewal and tumorigenesis of GSCs through regulation of FOXM1 and ADAM19, respectively. **(B)** Visvanathan et al. found that METTL3 is upregulated in GSCs. METTL3-mediated methylation in SOX2 mRNA through recruitment of HuR to enhance its stability in GSCs, the increased level of the SOX2 enhanced radiation resistance.

However, the diverse roles of m6A modification in GSC have been reported recently. Visvanathan et al. found a high level of entire METTL3-mediated m6A modification, associated with the maintenance of stem-like cells and the dedifferentiation of glioma cells (Visvanathan et al., 2018). Further experiments revealed that the pluripotency factor SOX2 was the m6A target of METTL3, and it was stabilized by recruiting Human antigen R (HuR) to m6A-modified SOX2 mRNA, resulting in decreased sensitivity to γ-irradiation (Visvanathan et al., 2018). In addition to the evidence that SOX2 was associated with radiation resistance in various cancers (Lee et al., 2015), the recruitment of HuR binding to m6A-modified transcripts was found to be preferential and global (Visvanathan et al., 2018), suggesting that other target genes of m6A modification may be involved in the regulation of irradiation sensitivity. These findings suggested that mRNA m6A levels seem opposite, predicting the diverse targets and functions of m6A modification in different processes of malignant tumors, such as tumorigenesis and radiation resistance, and suggesting the potential target role of m6A modification for the treatment of GSCs (Figure 4).

#### Colorectal Cancer (CRC)

Colorectal cancer (CRC), the second most common cause of cancer-related death in the United States, is generally treated with combined application of surgery, radiation, and chemotherapy (Siegel et al., 2020). However, with the recurrence of metastasis, it results in treatment failure, which is currently a major challenge. Colorectal CSCs (CCSCs) are reported to be the main causes of recurrence and metastasis in CRC patients (Wang et al., 2020). Since (Dalerba et al., 2007) identified CSCs in CRC in 2007, great efforts have been made to explore the underlying mechanism of the regulation of these cells in CRC and revealed special molecular pathways involved in CCSCs regulation, such as the Wnt/β-catenin pathway (Ordonez-Moran et al., 2015) and Notch signaling (Jin et al., 2017).

The function of m6A modification in CCSCs has also raised concern among researchers. To explore the role of YTHDF1 in CRC, Bai et al. overexpressed YTHDF1 in CRC and found that YTHDF1 can promote the tumorigenicity and xenograft tumor growth of cells in CRC *in vitro* and *in vivo*, respectively (Bai et al., 2019). Further investigation verified that overexpression of the reader protein YTHDF1 promoted colonosphere formation and self-renewal, thought inhibiting Wnt/β-catenin pathway activities in cells in CRC, while knockdown the expression of YTHDF1, inhibited colonosphere self-renewal while enhancing their differentiation (Bai et al., 2019). Although research is limited, the regulatory function of YTHDF1 in CCSCs evokes further investigations on the regulation of CSCs activities and their therapeutic targets for CRC patients.

#### Osteosarcoma

Osteosarcoma is a malignant bone tumor that has a high prevalence in adolescents and children, with a high mortality rate (Schneiderman et al., 1984). Although osteosarcoma is potentially initiated from a single cell as a monoclonal disease, the quick development of a polyclonal disease position it as one of the most complex cancers in terms of molecular aberration (Brown et al., 2017). Gibbs et al. (2005) first identified and reported osteosarcoma stem cells (OSCs) based on the expression of Oct 3/4, Nanog, and STAT3 in bone sarcoma cells, serving as potential targets for selective noncytotoxic therapy in bone sarcoma patients, which are rather resistant to current therapeutic protocols. OSCs play a central role in chemoresistance and in metastasis, which is the main cause of cancer-related death in patients with osteosarcoma (Yan et al., 2016).

Recently, the m6A modification and gene expression differences in OSCs were detected through m6A MeRIP-seq and RNA-seq and, and it was found that m6A-related enzymes, METTL3, METTLE14, and ALKBH5, were abnormally expressed in OSCs (Wang et al., 2019b). Importantly, the differentially methylated genes were enriched in signaling pathways regulating the pluripotency of stem cells and correlated with the poor prognosis in patients with osteosarcoma (Wang et al., 2019b). The m6A modification may be a breakthrough mechanism to improve the treatment of osteosarcoma and provide a fundamental contribution to the search for new therapeutic targets for OS (Wang et al., 2019b).

## Implications for Cancer Therapies

### The Treatment Strategies Targeting CSCs

As CSCs in malignant tumors provide a new therapeutic strategy for cancer treatment, four main CSC-targeted therapies directed at stem cell fate regulation are currently under development and investigation (Ahmad and Amiji, 2017; Pan et al., 2018).

(1) Antibodies targeting surface markers of CSCs. Based on the identification of specific surface markers for CSCs, such as CD34^+^/CD38^–^, CD33, and CD44^+^/CD24^–^ (Al-Hajj et al., 2003; ten Cate et al., 2010), antibodies against specific surface markers have been developed and even used in clinical settings. For example, as 80–90% of stem cells in AML express CD33, antibodies targeting CD33, such as gemtuzumab, became an important drug for the treatment of AML (Laing et al., 2017).

(2) Target drugs to CSC-related pathways. Series of abnormal activation of signaling pathways in CSCs, such as PI3K/Akt/mTOR, Wnt, and Notch, and other signaling pathways have been detected in different types of malignant tumors (Bertacchini et al., 2015; Takebe et al., 2015; Venkatesh et al., 2018); providing targeted therapy to these signaling pathways has also become an important therapeutic strategy. For example, the antitumor drugs rapamycin and everolimus, targeting the PI3K/Akt/mTOR signaling pathway, have been evaluated in the treatment of leukemia; however, further clinical studies are required (Bertacchini et al., 2015).

(3) Inducing the differentiation of CSCs. Compared with normal cells, the differentiation of CSCs is either abnormal or blocked (Pan et al., 2018). Therefore, inducing the differentiation and maturation of CSCs provides a useful and potential method to block their ability to self-renewal and effectively inhibit tumor growth (Han et al., 2015). Presently, 90% of the patients with acute promyelocytic leukemia were completely relieved by all-trans vitamin A acid-induced differentiation (Ohno et al., 2003).

(4) Changing the microenvironment of CSCs (Prasetyanti and Medema, 2017). It was revealed that the abnormal microenvironment transforms normal stem cells into CSCs, leading to the formation of malignant tumors (Liu and Fan, 2015). Therefore, restoring the tumor microenvironment to the normal one is particularly important to provide the potential of reversing CSC differentiation.

### Targeting CSC Therapies Associated With m6A Enzymes

Rhein is the first identified natural inhibitor of FTO (Chen et al., 2012). Niu et al. (2019) found that Rhein can inhibit breast cancer cell proliferation, colony formation, and metastasis *in vitro* and *in vivo*. However, the activity and specificity of these FTO inhibitors are relatively poor, and their mechanism of action has not been fully studied. MA2, the ethyl ester form of meclofenamic acid (MA), was recently identified as a selective inhibitor of FTO (Huang et al., 2015). MA2 was used in the treatment of GSCs and was effective in the *in vitro* and *in vivo* experiments, while the RNA m6A demethylases ALKBH5 and FTO enhanced self-renewal and tumorigenesis of GSCs (Cui et al., 2017). In GSC-grafted animals, MA2 suppressed glioblastoma progression and prolonged the lifespan of GSC-grafted animals (Cui et al., 2017).

Therefore, more effective FTO inhibitors need to be developed for clinical application. Recently, Huang et al. reported two new small molecule inhibitors of FTO, namely, FB23 and FB23-2, directly binding to FTO and specifically inhibiting the activity of m6A demethylase of FTO, finally resulting in the suppression of AML cell proliferation (Huang et al., 2019). Because of the reported oncogenic role of FTO in AML (Li Z. et al., 2017), their inhibitors such as FB23 and FB23-2 are expected to have a potential treatment effect in AML patients, and future potential for use in the clinic. However, drugs targeting m6A modification of CSCs in malignant tumors are limited, and further investigations are needed to explore potential targets and drugs in this field.

## Summary and Perspectives

Cancer stem cells, with self-renewal and tumorigenesis abilities, are the major cause for tumor recurrence and chemotherapy resistance. However, the underlying mechanisms have not been fully elucidated. Recent studies revealed the regulating role of m6A in the differentiation of CSCs. The present review focused on this field to review the function and regulating role of m6A modification in the differentiation of CSCs, especially to explore the potential mechanism underlying the determination of their fates. Currently, inhibitors of FTO and ALKBH5 can be used as candidates for anticancer drug development; especially to inhibit the growth of cancer cells by manipulating their m6A modification levels. Although these inhibitors have not been tested in clinical trials yet, they provide more possibilities for early diagnosis and treatment of cancer.

## Author Contributions

H-TW contributed conception and design of the study and revised the original manuscript critically. YX, JL, and H-TW organized the database, searched the literature, and structured and drafted the manuscript, figures, and table carefully. W-JC, Q-QY, W-TC, and C-LL organized the database and partially drafted the manuscript carefully. All authors contributed to manuscript revision, and read and approved the submitted version.

## Conflict of Interest

The authors declare that the research was conducted in the absence of any commercial or financial relationships that could be construed as a potential conflict of interest.
